# Gene-editing cholesterol trial paves way for groundbreaking treatment

**Published:** 2023

The treatment of a handful of patients with severe heart disease, who volunteered for an experimental cholesterollowering treatment using gene editing, has paved the way for the potential transformation of preventive cardiology, say experts. The patients, who suffered from heart attacks and pain, had been unable get their cholesterol as low as cardiologists recommended, despite trying all available medications. So they volunteered for the experimental treatment that was unlike anything tried in patients before.

The New York Times reports that the result, released by the company Verve Therapeutics of Boston at a meeting of the American Heart Association, showed that the treatment appeared to reduce cholesterol levels markedly in patients and that it appeared to be safe.

The trial involved only 10 patients, with an average age of 54 years. Each had a genetic abnormality, familial hypercholesterolaemia, which affects around 1m people in the United States.

But the findings could also point the way for millions of other patients around the world who are contending with heart disease, which remains a leading cause of death. And while more trials in a broader range of patients will need to be carried out, gene editing experts and cardiologists said the treatment could transform preventive cardiology.

‘Even for seasoned veterans of this field like me, this is a day we will look back on,’ said Fyodor Urnov, a gene editor at the Innovative Genomics Institute in Berkeley, California. ‘It’s like crossing a Rubicon, in a good way. This is not a small step. It is a leap into new territory.’

Impressed with the data and the potential, pharmaceutical giant Eli Lilly has paid $60m to collaborate with Verve Therapeutics and opted to acquire additional rights to Verve’s programmes for an additional $250m. If the editing continues to look promising, Eli Lilly expects to help with larger studies.

‘Until now, we thought of gene editing as a treatment we should reserve for very rare diseases where there is no other treatment,’ said Dr Daniel Skovronsky, Eli Lilly’s chief scientific and medical officer. ‘But if we can make gene editing safe and widely available, why not go after a more common disease?’

The study was led by Dr Sekar Kathiresan, chief executive of Verve. Patients received a single infusion of microscopic lipid nanoparticles containing within them a molecular factory to edit a single gene in the liver, the site of cholesterol synthesis. The gene, PCSK9, raises levels of low-density lipoprotein (LDL) cholesterol, the bad kind. The plan was to block it.

The little lipid spheres were carried through the blood directly to the liver. They entered the liver cells and opened up, revealing two molecules. One instructs DNA to make a gene editing tool, and the other is a guide to take the editing tool to the gene that needs editing.

The treatment ‘is almost like science fiction’, said Dr Martha Gulati, director of preventative cardiology at the Smidt Heart Institute of Cedars-Sinai Medical Centre in Los Angeles and president of the American Society for Preventive Cardiology, who was not involved in the trial.

The gene editing tool acts like a pencil and an eraser. The eraser wipes out one letter of the target gene, and the pencil writes in a new one, turning off PCSK9. The goal: a single cholesterol-lowering treatment that results in lifelong protection from heart disease. Patients received varying doses. LDL levels in the three who received the highest doses fell by 39 to 55%, enough to get them towards their cholesterol goal.

In the study, those who received the higher doses had flu-like symptoms for a few hours. Two patients had serious adverse events that the study’s independent data safety and monitoring board deemed a result of their underlying severe heart disease. The board advised the researchers not to stop the study.

One patient had a fatal cardiac arrest five weeks after receiving the infusion. An autopsy showed that several of his coronary arteries were blocked. The other patient had a heart attack the day after the infusion. It turned out that he had been having chest pain before receiving the infusion but had not reported it. If the investigators had known, he would not have received the treatment.

In a way, the treatment is a culmination of studies that began a decade ago when researchers discovered rare but healthy individuals with cholesterol levels that seemed impossibly low. The reason was that their PCSK9 gene was mutated and no longer functioned. As a result, these people were protected from heart disease.

That led to the development of antibodies to block the gene. Patients inject themselves with the antibodies once a week. Then came a twice-yearly RNA injection that prevents PCSK9 from being made. It seemed possible that those treatments, as well as statins for those whose cholesterol is more easily controlled, could help solve the heart disease problem.

But heart disease persists as a killer. Even after people are diagnosed with heart disease, less than 60% of all patients take a statin. Only a quarter take one of the more effective, high-intensity statins, Gulati said.

‘Patients take it initially, and then they forget or decide they don’t need it,’ she said. ‘That happens more than you’d think.’

Dr Michelle O’Donoghue, a cardiologist at Brigham and Women’s Hospital, said that because patients so often do not take their pills or injections, ‘there is a lot of interest, through gene editing, of a one and done – a single treatment and a lifetime response’.

Family history was the inspiration for Kathiresan at Verve Therapeutics. His uncle and grandmother died of heart attacks. His father had a heart attack at 54 years. And then, on 12 September 2012, his 42-year-old brother returned from a run dizzy and sweaty. He was having a heart attack. He died nine days later. At that moment, Kathiresan said, he vowed to find a way to prevent what had happened to his brother from happening to anyone else.

Of course, even if gene editing works, applying it to young people with heart risk is well into the future. But, Gulati said, early gene editing of younger patients with genetically high cholesterol levels might prevent arteries from hardening. ‘It could be an incredible medicine,’ she said.

This all depends on success and safety of the gene editing and on its effects lasting. The first patient was treated just six months ago. But a previous study in monkeys lasted two and a half years, and the results of the gene editing persisted.

Urnov, who said he has a genetic risk for heart disease, is optimistic for himself and his six-year-old daughter. ‘I honestly cannot wait for this medicine to become available for heart disease prevention,’ he said. ‘I love the idea of having gene editing as a vaccine for the prevention of heart disease.’ 
